# Characterization of two myostatin genes in pufferfish *Takifugu bimaculatus*: sequence, genomic structure, and expression

**DOI:** 10.7717/peerj.9655

**Published:** 2020-08-03

**Authors:** Yinzhen Sheng, Yulong Sun, Xin Zhang, Haifu Wan, Chengjie Yao, Keying Liang, Leibin Li, Bo Liu, Jianxing Zhong, Ziping Zhang, Yilei Wang

**Affiliations:** 1 Fisheries College, Jimei University, Xiamen, China; 2 College of Animal Science, Fujian Agriculture and Forestry University, Fuzhou, China; 3 Fisheries Research Institute of Fujian, Xiamen, P.R. China; 4 Key Laboratory of Marine Biotechnology of Fujian Province, Institute of Oceanology, Fujian Agriculture and Forestry University, Fuzhou, China

**Keywords:** *mstn1*, *mstn2*, Sequence, Genomic structure, Expression pattern, *Takifugu bimaculatus*

## Abstract

Myostatin (MSTN) is a negative regulator of muscle growth, which restrains the proliferation and differentiation of myoblasts. To understand the role of two *mstn* genes of *Takifugu bimaculatus*, the full-length cDNAs of 1131 bp *Tbmstn1* and 1,080 bp *Tbmstn2* were obtained from the *T. bimaculatus*’ genomic database, which encodes 376 and 359 amino acids, respectively. The results of qRT-PCR showed that *Tbmstn1* was expressed in the eye, kidney, spleen, skeletal muscle, gill, and brain, and the expression level in the skeletal muscle was extremely significantly higher than in other examined tissues. *Tbmstn2* was expressed in the skin, skeletal muscle, gill, and brain, and had the highest expression in the skeletal muscle, followed by expression in the brain. Meanwhile, in different stages of embryonic development, the expression of *Tbmstn1* started from the gastrula stage. Its expression in the eye-pigment formation stage and hatching stage was significantly higher than that in other stages. The *Tbmstn2* was expressed in all examined embryonic stages with different levels, and the highest expression was detected in the eye-pigment formation stage. These results suggested that *Tbmstn1* and *Tbmstn2* may involve in the development of skeletal muscle, and *Tbmstn2* may be related to the formation of nervous system.

## Introduction

*Takifugu bimaculatus*, a kind of pufferfish, belongs to Tetraodontiformes, Tetraodontidae, which is a carnivorous, demersal fish and prefers to live near coral reefs. It is widely distributed from the south China sea to the southern part of the yellow sea (Xie, 2017; Zhong, 2003). Pufferfish are famous for their puffing behavior, powerful toxins in the internal organs, and edible muscle (Kato et al., 2005). Because of its high economic and nutritional value from its excellent meat quality, *T. bimaculatus* has been cultured commercially in Fujian for a long time. Understanding the molecular information of growth regulation genes of *T. bimaculatus* can provide a useful guide for its aquaculture.

Cell growth and development are achieved through the synergistic action of a series of growth factors. Depending on their biological functions, these growth factors can be classified into several types, some of which promote cell growth and proliferation, while others inhibit them. Some growth factors are concentration-dependent, for example, transforming growth factors (TGFs) play a promoting role in growth at appropriate concentrations and an inhibiting role at inappropriate levels. Their transformation ability is jointly completed by two components (named TGF-*α* and TGF-β, respectively), which differ significantly in molecular composition, receptor structure, and biological effects (Anzano et al., 1983). Previous studies have found that TGF-β is a large family of proteins consisting of many signaling factors, collectively referred to as the TGF-β superfamily. Members of this superfamily include TGF-βs, bone morphogenetic proteins (BMPs), growth differentiation factors (GDFs), glial cell-derived neurotrophic factors (GDNFs), activins/inhibins, mullerian inhibitory substance (MIS), and macrophage inhibitory cytokine-1 (MIC-1). TGF-β superfamily proteins play an essential role in the formation and growth of muscle tissue (Chin et al., 2004; Massague, 1998; Piek, Heldin & Ten, 1999). They are a group of cytokines in the form of peptides, and their members have many similar features: (1) at the beginning of synthesis, they are a precursor molecule with a large molecular weight, including a hydrophobic N-terminal that contains a secreted signal peptide and leading peptide, which can be used to cross the endoplasmic reticulum; (2) the precursor molecule is cleaved at the protease processing site consisting of four amino acids (RSRR), and then a mature polypeptide (also known as subunit) of about 110 to 140 amino acids was released; (3) The C-terminus is a biologically active region containing seven or nine conserved cysteines, which forms a biologically active dimer by intermolecular disulfide bonds. When these factors are just secreted, they are a non-bioactive entity with a leader peptide, which requires a special mechanism to cleave the precursor protein and the coordination of other growth factors to make them have biological activity. Therefore, the TGF-β superfamily has more complex biological functions (Cheifetz et al., 1987; Massague, 1998).

Myostatin (MSTN) is also called growth differentiation factor-8 (GDF-8). Grobet et al. (1997) first discovered it when they studied Belgian blue cattle. Mcpherron & Lee (1997) confirmed that it is a member of TGF-β superfamily and is a conserved negative regulator of myocyte growth in mammals (Mcpherron, Lawler & Lee, 1997). As a negative regulator of muscle growth and differentiation, MSTN restrains the proliferation and differentiation of myoblasts by inhibiting the transcriptional activity of the positive regulatory factor, mitogenic determination gene (MyoD) (Kerr, Roalson & Rodgers, 2005; Ríos et al., 2002). In domesticated species, natural mutations of *mstn* gene have been found, and improve the yields of cattle (Tong et al., 2015). In contrast to the almost muscle-specific expression of the single mammalian *mstn* gene, teleost fish possess at least two *mstn* genes to be expressed in both muscular and non-muscular tissues (Ostbye et al., 2007). It is generally believed that the two or more copies of the *mstn* gene in teleost fish are the result of polyploidization (Maccatrozzo et al., 2001a; Ostbye et al., 2010; Stinckens, Georges & Buys, 2011; Xu & Chen, 2008).

The identification of a strong negative regulator of muscle mass, such as *mstn* offers the perspective to control muscle mass by targeting the MSTN pathway (Gabillard et al., 2013). In an aquaculture point of view, controlling MSTN activity may help to improve the management of muscular growth of teleost fish, as well as to define a new strategy to control both meat quantity and quality. Up to date, many sequences of *mstn* genes have been identified in teleost fish, such as common carp *Cyprinus carpio* (Li et al., 2007), zebrafish *Danio rerio* (Helterline et al., 2007; Xu, Wu & Du, 2003), grass carp *Ctenopharyngodon idellus* (Zheng et al., 2015), yellowcheek carp *Elopichthys bambusa* (Yu et al., 2010), cyprinid loach *Misgurnus anguillicaudatus* (Peng et al., 2007), rainbow trout *Oncorhynchus mykiss* (Garikipati et al., 2007; Rescan, Jutel & Rallière, 2001), Atlantic salmon *Salmo salar* (Ostbye et al., 2010), Mandarin fish *Siniperca chuatsi* (Zhang, Zhao & Deng, 2010), tilapia *Oreochromis mossambicus* (Rodgers et al., 2001), gilthead seabream *Sparus aurata* (Maccatrozzo et al., 2001b), large yellow croaker *Larimichthys crocea* (Liu et al., 2014), channel catfish *Ictalurus furcatus* (Kocabas et al., 2002), Japanese flounder *Paralichthys olivaceus* (Xu & Chen, 2008). In pufferfish, there are few studies on the *mstn* gene. In this study, we identified and characterized *mstn1* and *mstn2* genes in *T. bimaculatus* (*Tbmstn1* and *Tbmstn2*). We also analyzed their expression patterns in detail by quantitative real-time PCR (qRT-PCR). This research seeks to provide some valuable references for further understanding the roles of *mstn* genes play in the fish.

## Materials & Methods

All of the study design and fish experiments were conducted in accordance with the guidelines of the Animal Administration and Ethics Committee of Jimei University (Permit No. 2011-58; 2011-59).

### Animals and tissues

*T. bimaculatus* fish were purchased from the Dajing Takifugu Breeding Farms in Zhangzhou, Fujian Province, China. Four live adult fish were anesthetized (MS-222 at 30 µg/mL) and then killed by severing the spinal cord. Eye, fin, liver, gallbladder, kidney, spleen, skin, heart, intestine, skeletal muscle, gill, and brain were sampled and stored immediately in RNAlater overnight at 4 °C, and then kept at −20 °C for RNA isolation. Parental fish was injected with lutein releasing hormone (LRH) and human chorionic gonadotrophin (HCG) for spawning inducing (Li et al., 2016; Mylonas & Zohar, 2007). Embryos reared in the seawater at 25 °C. Surviving animals after the experiment were returned to Dajing Takifugu Breeding Farms.

Embryos were collected according to developmental stages, including the two-cell stage, blastula stage, gastrula stage, neurula stage, somite appearance stage, eye lens formation stage, muscular activity stage, heart pulsation stage, eye pigment formation stage and hatching stage (Chen, 2005). Some of them were stored immediately in RNAlater overnight at 4 °C, and then kept at −20 °C for RNA isolation, and the rest were fixed in 4% paraformaldehyde-PBS fixation buffer (PFA) overnight at 4 °C, and then rinsed with 1 × PBS, dehydrated in gradient dilute methanol (70, 80, 90, and 100% methanol), finally kept at −20 °C until use for WMISH.

### RNA isolation and cDNA synthesis

Total RNA was extracted from each tissue and embryo of *T. bimaculatus* with Total RNA Extraction Kit (Promega, Shanghai, China) following the manufacturer’s protocol. The total RNA quality was assessed by agarose gel electrophoresis and Nanodrop 2000 (Thermo Scientific, USA), and then treated with DNase I (Promega, Shanghai, China) to remove the potentially contaminated DNA. Subsequently, for qRT-PCR, 1 µg of total RNA of each tissue and embryo were used to synthesize the first-strand cDNA with random hexamers using the M-MLV reverse transcriptase for RT-PCR amplification (Promega, Shanghai, China). The synthesized first-strand cDNAs were diluted and stored at −80 °C.

### Molecular cloning of *Tbmstn1* and *Tbmstn2*

Based on the genomic database from Professor Xu Peng, Xiamen University, both *mstn* paralogs were identified and named *Tbmstn1* and *Tbmsth2*, respectively. Then, two pairs of primers (*mstn1*-F/R; *mstn2*-F/R, Table 1) were designed using Primer 5.0 software to amplify the open reading frame (ORF) sequences to ensure the accuracy of the genomic result.

**Table 1:** The primers used in the present study.

**Gene**	**Primer**	**Sequence (5′–3′)**
*mstn1*	F	ATGCAACTGTCTCCGAGCAT
*mstn1*	R	AGACATCCACAACGGTCCAC
*mstn1*	qRT-PCR-F	GGATGAAAGAGGCTCCGAACA
*mstn1*	qRT-PCR -R	ACGTCCCTGTTGTCATCGC
*mstn1*	T7-F	AGCTCCTCGACCAGTACG
*mstn1*	T7-R	GTCACGGCCAAGTCTTTT
*mstn2*	F	TGTGCTGACCGTCGTCTCT
*mstn2*	R	GGATTTCTTTTCCTCGCTGGG
*mstn2*	qRT-PCR -F	GGAACAGGCTCCCAACATCA
*mstn2*	qRT-PCR -R	CTCCACCCTGGGATCGTACT
*mstn2*	T7-F	GCCACTAAGCCTAATCCC
*mstn2*	T7-R	ATCCAGTCCCATCCAAAC
*β-actin*	F	CAATGGATCCGGTATGTGC
*β-actin*	R	CGTTGTAGAAGGTGTGATGCC

### Sequences analysis

Nucleotide sequence homology analysis was performed by BLAST software (http://blast.ncbi.nlm.nih.gov/Blast.cgi). The ORFs were analyzed with ORF Finder (NCBI, Bethesda, MD, USA) (http://www.ncbi.nlm.nih.gov/projects/gorf/orfig.cgi). The theoretical amino acid composition, isoelectric point and molecular weight (Mw) were computed using the Expasy ProtParam Tool (http://web.expasy.org/protparam/). The amino acid sequences were submitted to predict the signal sequence with the SignalP 5.0 server (Technical University of Denmark, Lyngby, Denmark) (http://www.cbs.dtu.dk/services/SignalP/). N-glycosylation sites (N-X-S/T) were predicted with the NetNGlyc1.0 Server (http://www.cbs.dtu.dk/services/NetNGlyc/), phosphorylation sites were predicted with the NetPhos 2.0 Server (http://www.cbs.dtu.dk/services/NetPhos/). Protein domains were predicted by SMART research tool (http://smart.embl.de/). Vertebrates’ MSTNs were searched with the BLAST program. The phylogenetic tree was constructed by using the bootstrap neighbor-joining method in MEGA 8.0 software (http://www.megasoftware.net) and the bootstrap values were replicated 1,000 times. Vertebrate *mstns* were searched on NCBI genome database (https://www.ncbi.nlm.nih.gov/genome/), followed by analysis online using Splign (https://www.ncbi.nlm.nih.gov/sutils/splign/splign.cgi).

### qRT-PCR

The gene-specific primers for qRT-PCR (Table 1) were designed by the Primer 5.0 software based on the above-cloned sequence. *β*-*actin* was selected as the reference gene (Table 1) (Yan et al., 2018). And we sequenced PCR products of each primer pair to ensure that the correct target was amplified. The reverse transcription products of each sample (tissue or embryo) for qRT-PCR were properly diluted as the templates. The tissue with four biological replicates and the embryo with three biological replicates were assayed. Three technical replicates were performed in all samples. qRT-PCR was performed with the SYBR Green PCR Master Mix (Applied Biosystems) and analyzed by generating a melting curve in the Light Cycler 480 system. Each reaction of 10 µL qPCR mixture comprised 4.5 µL cDNA (10 ng/µL), 0.25 µL 10 µM forward primer, 0.25 µL 10 µM reverse primer, 5 µL SYBR Green PCR Master Mix. All qPCR reactions were as follows: 94 °C for 10 min, followed by 40 cycles of 95 °C 30 s, 60 °C 30 s, and 72 °C 30 s, finally 4 °C to terminate the reaction. The cDNA concentration represents the RNA equivalent from the cDNA synthesis reaction. CT values <40 were acceptable (Bustin et al., 2009). The data were expressed as the mean of RQ value (2 − ΔΔCT) (ΔCT = CT of target gene minus CT of *β-actin*, ΔΔCT = ΔCT of any sample minus calibrator sample) and analyzed with SPSS version 20.0 for one-way analysis of variance (one-way ANOVA) and bonferroni correction of post-test. The statistically significant differences were shown at *p* < 0.05, and the most significant differences were shown at *p* < 0.01.

## Results

### Molecular characterization of *Tbmstn1* and *Tbmstn2*

**Figure 1:**
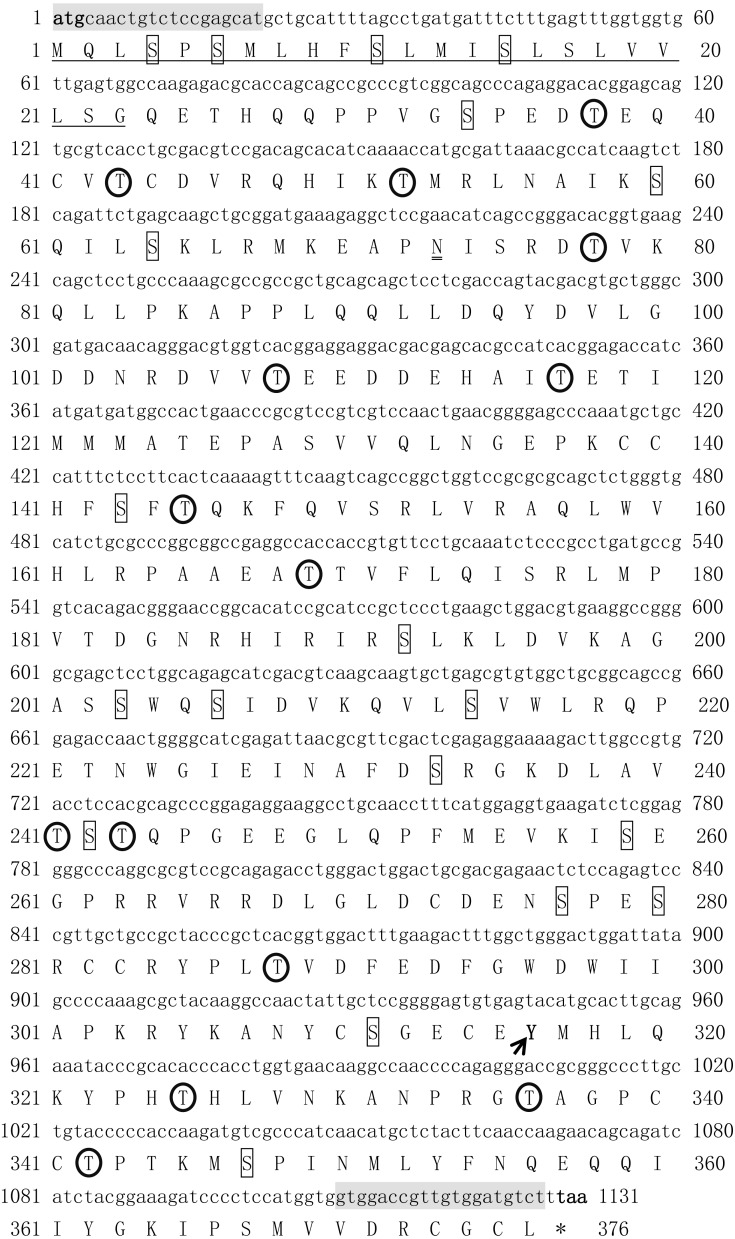
The cDNA and deduced amino acid sequence of *Tbmstn1* from *Takifugu bimaculatus*. The initiation codon (atg) and the stop codon (tag) are all characterized in bold. The glycosylation sites are indicated by double underline. The signal peptide sequence is indicated by an underline. The serine phosphorylation sites, the threonine phosphorylation site, and the tyrosine phosphorylation sites are indicated by boxes, arrows and circles respectively. The primers used for cDNA cloning are indicated by gray background.

**Figure 2:**
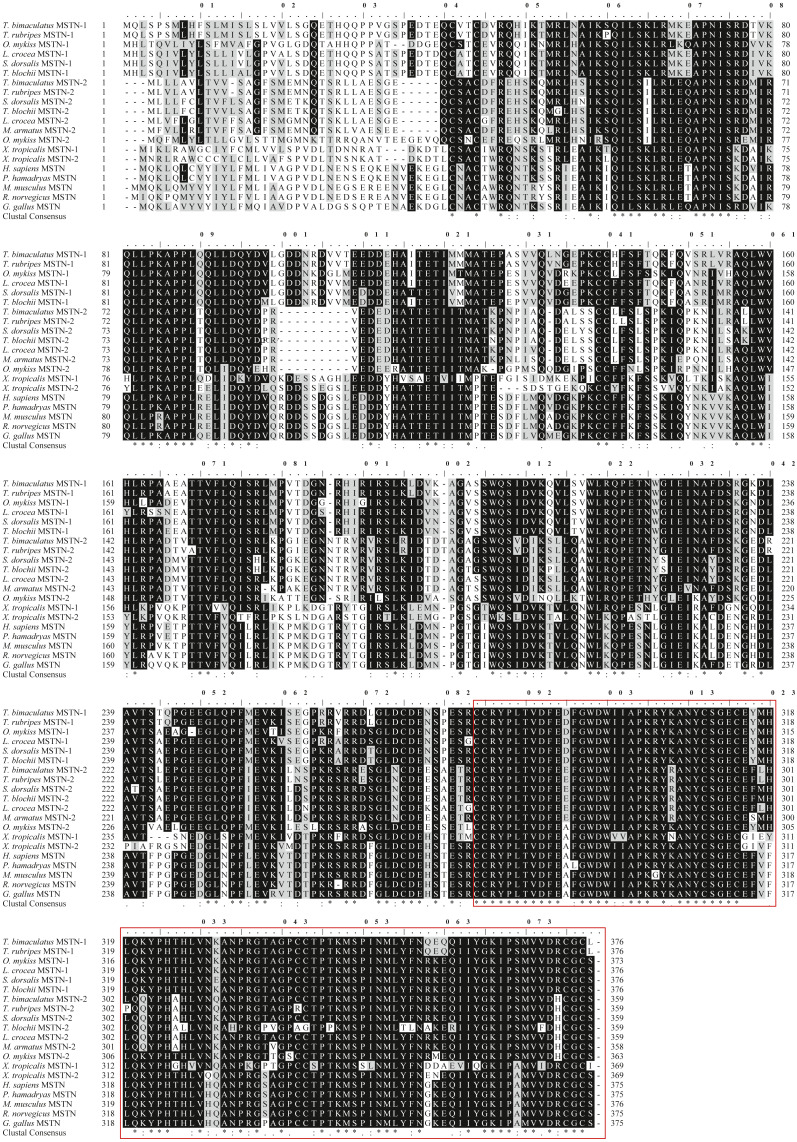
Protein multiple alignment of MSTN-1 and MSTN-2 between *T. bimaculatus* and other known vertebrates. Sequences shaded in black and grey represent conservative and semi-conservation amino acid substitutions, respectively. The GenBank accession numbers of the selected MSTN-1 and MSTN-2 proteins are shown as follows: *T. bimaculatus* MSTN-1 (MN733728), *T. rubripes* MSTN-1 (NM_001032672.1), *O. mykiss* MSTN-1 (AAK71707.1), *L. crocea* MSTN-1 (AAW34055.1), *Seriola lalandi dorsalis* MSTN-1 (AZQ19980.1), *Trachinotus blochii* MSTN-1 (AYA21607.1), *T. bimaculatus* MSTN-2 (MN733729), *T. rubripes* MSTN-2 (NM_001032671.1), *S. dorsalis* MSTN -2 (XP_023285554.1), *T. blochii* MSTN-2 (AXR95004.1), *L. crocea* MSTN-2 (ADY18336.1), *Mastacembelus armatus* MSTN-2 (XP_026157270.1), *O. mykiss* MSTN-2 (ABD91702.1), *X. tropicalis* MSTN-1 (XP_002931542.1), *X.tropicalis* MSTN -2 (XP_002931568.1), *H. sapiens* MSTN (AAH74757.2), *Papio hamadryas* MSTN (AAB86686.1), *M. musculus* MSTN (AAI05675.1), *Rattus norvegicus* MSTN (AAB86691.1), *G. gallus* MSTN (AAK18000.1). TGF-β domain is indicated by a red box.

The open reading frame (ORF) of *Tbmstn1* is 1131 bp encoding a polypeptide of 376 amino acids with a predicted molecular mass of 42.6 kDa and a theoretical pI of 6.38. There are 35 phosphorylation sites (Ser:20, Thr:14, Tyr:1) and 1 glycosylation site. The first 23 amino acids (Cleavage site between pos. 23 and 24: LSG-QE) were identified as a signal peptide (Fig. 1). The highly conserved TGF-β domains of the TGF-β superfamily was found at 282–376 residues of *Tb*MSTN1 (Fig. 2). The *Tbmstn1* cDNA sequence has been submitted to the NCBI GenBank (accession number: MN733728).

**Figure 3:**
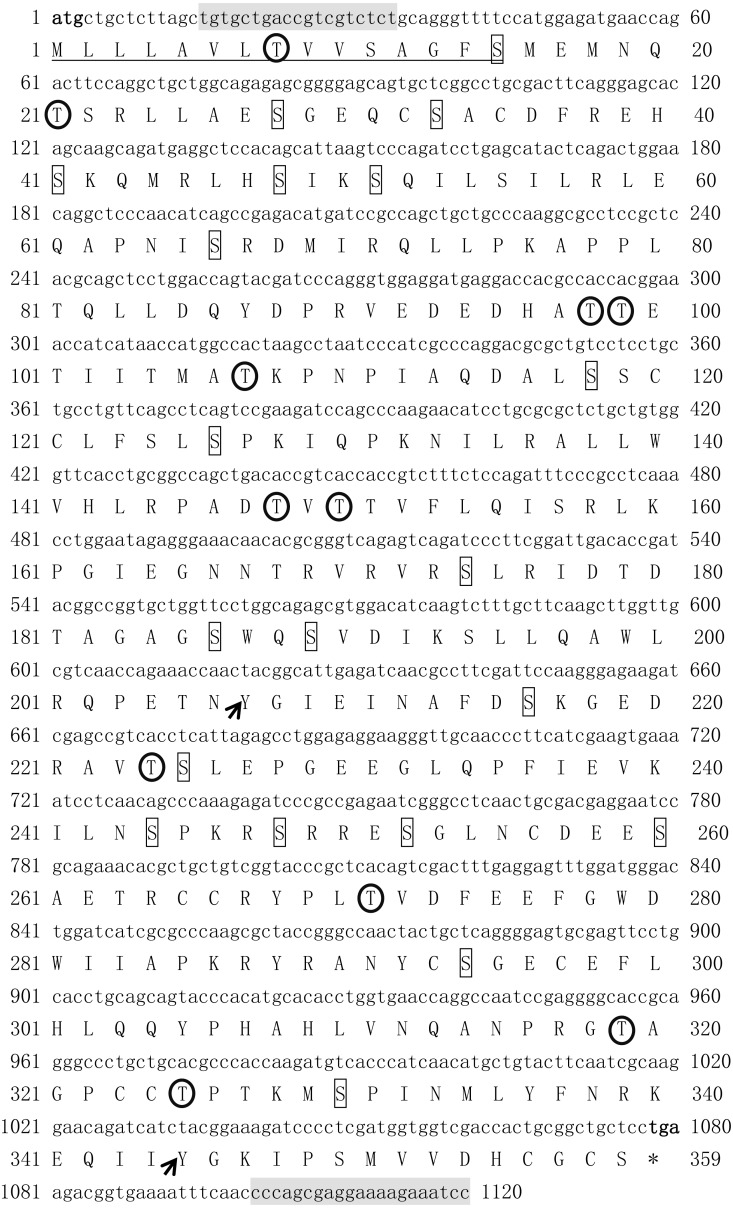
The cDNA and deduced amino acid sequence of *Tbmstn2* from *Takifugu bimaculatus*. The initiation codon (atg) and the stop codon (tag) are all characterized in bold. The glycosylation sites are indicated by double underline. The signal peptide sequence is indicated by underline. The serine phosphorylation sites, the threonine phosphorylation site, and the tyrosine phosphorylation sites are indicated by boxes, arrows and circles respectively. The primers used for cDNA cloning are indicated by gray background.

The ORF of *Tbmstn2* is 1,080 bp encoding a polypeptide of 359 amino acids with a predicted molecular mass of 40.4 kDa and a theoretical pI of 6.47. There are 33 phosphorylation sites (Ser:20, Thr:11, Tyr:2) and 3 glycosylation sites. The first 15 amino acids (Cleavage site between pos. 15 and 16: GFS-ME) were identified as a signal peptide (Fig. 3). The highly conserved TGF-β domains of the TGF-β superfamily was found at 265-359 residues of *Tb*MSTN2 (Fig. 2). The *Tbmstn2* cDNA sequence has been submitted to the NCBI GenBank (accession number: MN733729).

### Genomic organization of *mstn* genes in vertebrates

**Figure 4:**
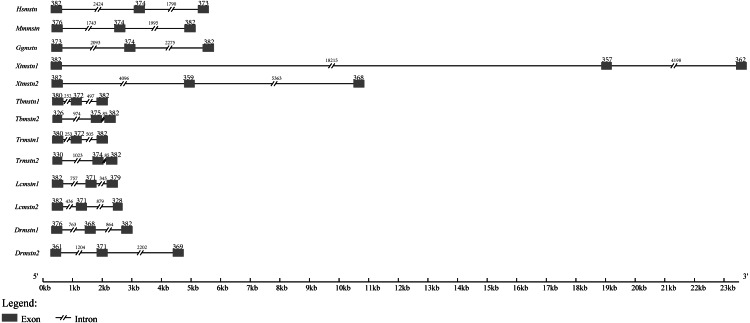
Schematic comparison of *Tbmstn1* and *Tbmstn2* genomic organization with other vertebrates *mstn* genes. The genomic organizations of *mstn* genes were compared among different species: *T. rubripes* (*Trmstn1* and *Trmstn2*), *L. crocea* (*Lcmstn1* and *Lcmstn2*), *D. rerio* (*Drmstn1* and *Drmstn2*), *Homo sapiens* (*Hsmstn*), *Mus musculus* (*Mmmstn*), *Gallus gallus* (*Ggmstn*) and *Xenopus tropicalis* (*Xtmstn1* and *Xtmstn2*). Exons and introns are shown by boxes and lines. The lengths in base pairs (bp) can refer to the scale below.

By searching other vertebrate genomic sequences of *mstn* genes in NCBI databases, the comparison of genomic organization among *T. bimaculatus*, *T. rubripe* s, *L. crocea*, *D. rerio*, *Homo sapiens*, *Mus musculus*, *Gallus gallus,* and *Xenopus tropicalis* was generated. The results showed that the genomic organization of *Tbmstn1* and *Tbmstn2* was composed of three exons and two introns, which was similar to that found in *T. rubripe* s, *L. crocea*, *D. rerio*, *H. sapiens*, *M. musculus*, *G. gallus* and *X. tropicalis* (Fig. 4). Although such a genomic arrangement was quite similar among the analyzed species, the length of the exons and introns was different from each other, especially the size of the intron. *Xtmstn1* had the longest intron1, and *Xtmstn2* had the longest intron2, while *Tbmstn1* had the smallest intron1, and *Tbmstn2* had smallest intron2 (Fig. 4).

### Homology analysis and phylogenetic analysis of MSTN-1 and MSTN-2

Multiple sequence alignment of MSTN-1 and MSTN-2 for homology analysis was performed among *T. bimaculatus*, other teleost, mammals, amphibians, and birds. The results showed that both MSTN-1 and MSTN-2 in teleost shared the high identity with other species, respectively, but the length of the encoded amino acid was different (Fig. 2). Meanwhile, *Tb*MSTN-1 shared the highest identity with *T. rubripes* MSTN-1 (99%), and *Tb*MSTN-2 shared the highest identity with *T. rubripes* MSTN-2 (98%). Also, *Tb*MSTN-1 and *Tb*MSTN-1 were highly divergent, sharing a relatively low amino acid sequence identity of 66%.

**Figure 5:**
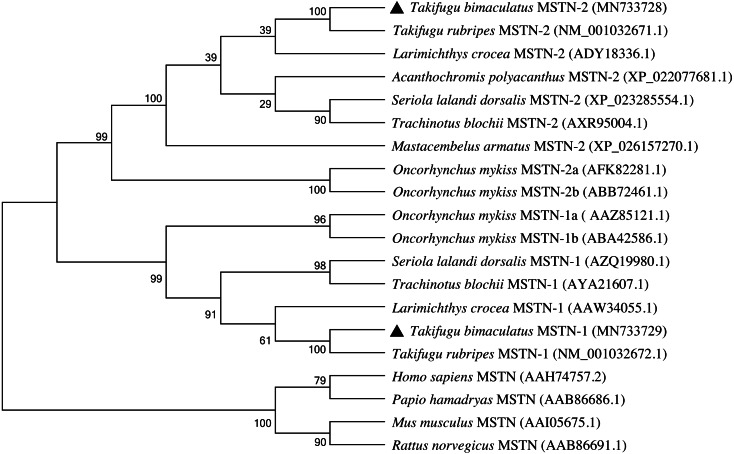
Phylogenetic tree of the MSTN-1 and MSTN-2 amino acid sequences between *T. bimaculatus* and other teleost and mammals. The GenBank codes of species are described in brackets. The *Tb*MSTN-1 and *Tb*MSTN-2 are marked by black triangle. The numbers near the node mean bootstrap values of 1,000 replicates.

A phylogenetic tree was constructed to elucidate the evolutionary relationship of MSTN-1 and MSTN-2 in vertebrates by the N-J method of MEGA 8.0 (Fig. 5). The analysis of the phylogenetic relationship indicated that the MSTN of mammals was clustered into one group, which was separated from teleost. The MSTN-1 of teleost and MSTN-2 of teleost were clustered into two subgroups respectively and then clustered together as a group. Among them, both *Tb*MSTN-1 and *Tb*MSTN-2 were clustered with *T. rubripes* into one small branch, respectively.

### Tissue expression analysis of *Tbmstn1* and *Tbmstn2*

**Figure 6:**
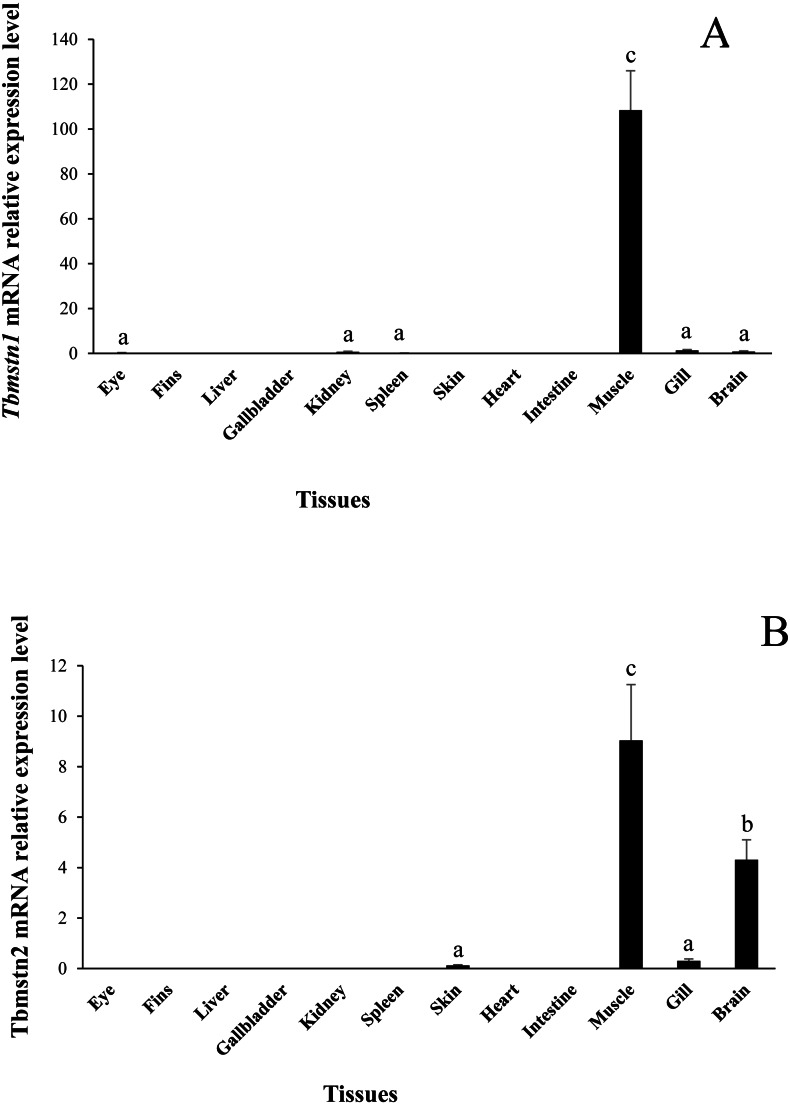
Expression of *Tbmstn1* and *Tbmstn2* mRNA in different tissues of *T. bimaculatus*. (A) qRT-PCR of *Tbmstn1*; (B) qRT-PCR of *Tbmstn2*; Each bar represented mean ± SEM (*n* = 4). The lowercase letters above the error bars signify the difference as follows: the same letters are no significant different (*p* > 0.05), the intervallic letters are extremely significantly different (*p* < 0.01). *Tb*-*β*-*actin* served as a reference gene.

The expression of *Tbmstn1* and *Tbmstn2* in *T. bimaculatus* tissues was analyzed by qRT-PCR. *Tbmstn1* was expressed in the eye, kidney, spleen, skeletal muscle, gill, and brain. Among them, the expression levels in the skeletal muscle were extremely significantly higher than in other examined tissues (*p* < 0.01), and the expression of *Tbmstn1* was low in other tissues with no significant difference (*p* > 0.05) (Fig. 6A). *Tbmstn2* was expressed in the skin, skeletal muscle, gill, and brain. The highest expression level was in the skeletal muscle (*p* < 0.01), next was in the brain (*p* < 0.05). The expression of *Tbmstn2* was low in other tissues with no significant difference (*p* > 0.05) (Fig. 6B).

### Embryo expression analysis of *Tbmstn1* and *Tbmstn2*

**Figure 7:**
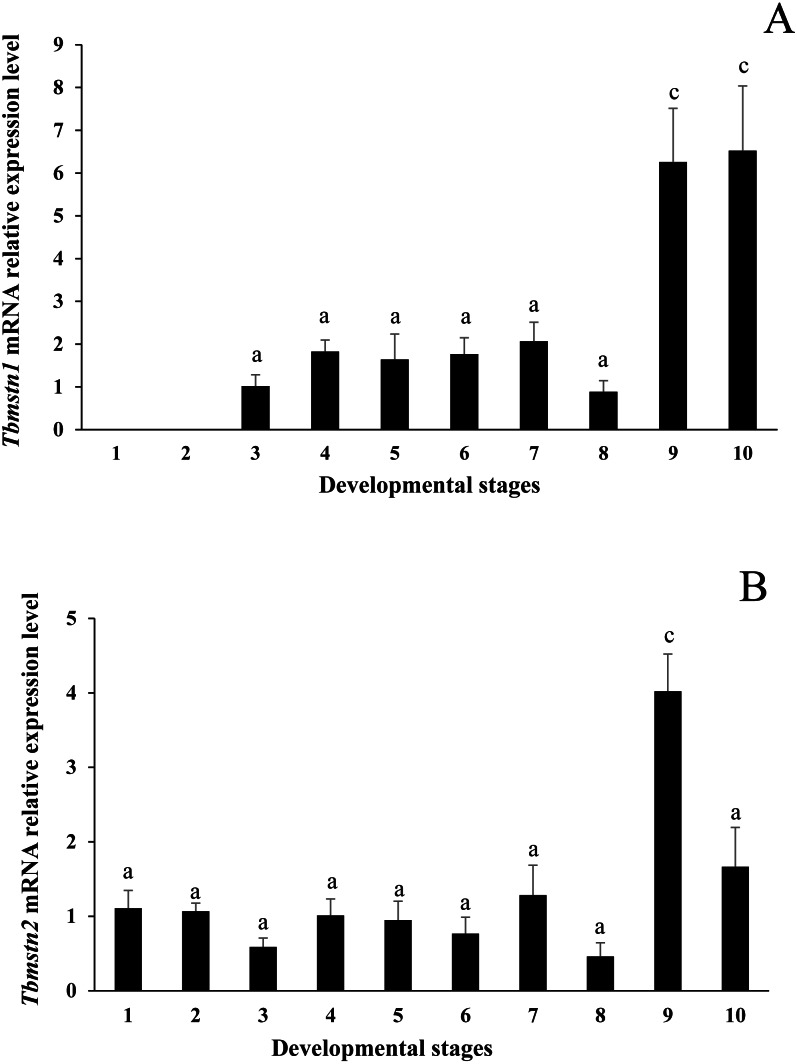
Expression of *Tbmstn1* and *Tbmstn2* mRNA in different embryonic stages of *T. bimaculatus*. (A) qRT-PCR of *Tbmstn1*; (B) qRT-PCR of *Tbmstn2*; Each bar represented mean ± SEM (*n* = 3). The lowercase letters above the error bars indicate the difference as follows: the same letters are no significant different (*p* > 0.05), the intervallic letters are extremely significantly different. *Tb*-*β*-*actin* served as a reference gene. Abbreviations: 1. two-cell stage; 2. blastula stage; 3. gastrula stage; 4. neurula stage; 5. somite appearance stage; 6. eye lens formation stage; 7. muscular activity stage; 8. heart pulsation stage; 9. eye pigment formation stage; 10. hatching stage.

The qRT-PCR results showed that the expression of *Tbmstn1* started from the gastrula stage. Its expression in the eye-pigment formation stage and hatching stage was significantly higher than that in other stages (*p* < 0.01) (Fig. 7A). The *Tbmstn2* was expressed in all examined embryonic stages, and the highest expression was detected in the eye pigment formation stage (*p* < 0.01) (Fig. 7B).

## Discussion

In mammals and birds, the *mstn* gene is present in a single copy and is mainly expressed in skeletal muscle (Gabillard et al., 2013; Xu & Chen, 2008). Due to the presence of multiple paralogous *mstn* genes in fish, the expression levels and distribution of *mstn* are different in different gene subtypes and different species (Roberts & Goetz, 2001; Rodgers et al., 2001). Helterline et al. (2007) found that there were two different *mstn* genes in zebrafish with significant differences in structure, expression pattern, and function. In this study, *Tbmstn1* and *Tbmstn2* encode a protein consisting of 376 and 359 amino acids, respectively, and *Tbmstn1* shares a low identity with *Tbmstn2* (66.85%). In grass carp, *mstn1* and *mstn2* also are highly divergent, sharing a relatively low amino acid sequence identity of 66% (Zheng et al., 2015). The phylogenetic analysis showed that teleost MSTN-1 and MSTN-2 were grouped into two clusters. These results indicated that different sequences in teleost MSTN-1 and MSTN-2 might lead to functional differentiation. On the other hand, multiple sequence alignment revealed that both MSTN-1 and MSTN-2 of teleost shared the high identity with those of vertebrates, respectively. Meanwhile, like other *mstn* genes in mammals, amphibians, birds, and teleost, both *Tbmstn1* and *Tbmstn2* genes consist of three exons and two introns, and their proteins all contain a highly conserved TGF-β domains of the TGF-β superfamily (Lee et al., 2016), revealing a remarkable structural conservation throughout evolution.

Unlike the mammalian *mstn* gene, which was mainly expressed in skeletal muscle, heart, and brain, the teleost fish *mstn* gene was expressed in muscle, eye, gill, spleen, intestine, brain, ovary, and testis (Rodgers et al., 2001). Moreover, the *mstn* expression of red muscle was higher than that of white muscle (Rodgers et al., 2001; Xu, Wu & Du, 2003). For example, in grass carp, *mstn1* had the highest expression in muscle, brain, and eye, followed by liver, pancreas, spleen, and heart *mstn2* was mainly expressed in the brain. It was also highly expressed in muscle, gonad, and eye (Pu et al., 2011). In zebrafish, *mstnb* (*mstn1*) was mainly expressed in the brain, heart, testis, and white muscle, and *mstna* (*mstn2*) was mainly expressed in the brain, spleen, and testis (Helterline et al., 2007). In Asian sea bass (*Lates calcarifer*), *mstn1* was mainly expressed in gill, muscle, and brain, whereas *mstn2* was mainly expressed in brain and gill (De Santis & Jerry, 2011). In large yellow croakers, *mstn1* was expressed in the liver, kidney, brain, intestine, and skeletal muscle, and *mstn2* was expressed in the spleen, brain, adipose, kidney, gill, eye, intestine, and liver (Liu et al., 2014; Xue et al., 2012). In our study, we found that the *Tbmstn1* was expressed in the eye, kidney, spleen, muscle, gill, and brain. The expression level in muscle was significantly higher than that in other tissues. The *Tbmstn2* was expressed in the skin, muscle, gill, and brain, and the highest expression occurred in muscle, followed by brain. All together, these data showed that *mstn1* in teleost fish was significantly expressed in skeletal muscles, which further indicated its important regulatory role in the growth and development of teleost fish muscle. In addition, these data clearly showed that skeletal muscle in teleost fish is not unique source of *mstn*. In fact, all known teleost fish *mstn* orthologs were expressed in the brain, especially *mstn2*, which was expressed at a high level in the brain. These indicate that the brain in teleost fish is important source of *mstn*, which may be involved in the development of the nervous system.

At the embryonic development stage, in zebrafish, the expression of the *mstnb* (*mstn1*) gene was low within 24 h of fertilization and then increased significantly with the development of the embryo, suggesting that the gene may be related to the development of embryonic segments. However, the expression of *mstna* (*mstn2*) gene was low at different stages of embryo development (Xu, Wu & Du, 2003). Using available public datasets of embryo-wide RNA-Seq experiments in zebrafish (https://www.ebi.ac.uk/gxa/experiments/E-ERAD-475) we found that both *mstn* genes were expressed at the larval stage of protruding mouth, with the highest expression at larval day 4. Interestingly, in *Gymnocypris przewalskii*, *mstn1* was expressed in every development stage, but *mstn2* could be detected from gastrulation and then increased until hatching (Tong et al., 2015). In tilapia and rainbow trout, both *mstn1* and *mstn2* transcripts were nearly undetectable during gastrulation (Garikipati, Gahr & Rodgers, 2006; Rodgers et al., 2001). In our study, the expression of *Tbmstn1* started from the gastrula stage. Its expression was significantly higher in the eye pigment formation stage and the hatching stage than that in other stages. The *Tbmstn2* was expressed in all examined embryonic stages with different levels. The highest expression was detected in eye pigment formation stage. Our results were inconsistent with the observation in grass carp, grass carp *mstn1* and *mstn2* were upregulated at the start of somitogenesis, and their levels continued to rise or were stable until hatching (Zheng et al., 2015). In large yellow croaker, both *mstn1* and *mstn2* transcript also could be detected in just-fertilized eggs (Liu et al., 2014). This difference may reflect the diverse expression patterns of *mstn* genes in different species.

The negative regulatory effect of *mstn* on muscle growth and development has been widely confirmed (Mcpherron, Lawler & Lee, 1997). In RNAi-mediated *mstn* silenced zebrafish, body weight gain was observed (Lee et al., 2009). In *mstn* gene-edited Channel catfish (*Ictalurus punctatus*), body weight gain also was observed (Khalil et al., 2017). In olive flounder (*Paralichthys olivaceus*), CRISPR/Cas9-mediated *mstn* disruption similarly enhanced muscle mass (Kim et al., 2019). These results showed that MSTN could inhibit skeletal muscle growth. Besides, Wang et al. (2017) found that both MSTN paralogs participate in the innate immune response using the CRISPR/Cas9 technique in zebrafish. Chiang et al. (2016) also revealed that MSTN deficiency results in suppression of the immune system using *mstn* mutated (*mstn*^-/-^) medaka generated from TALENs technology, a genome-editing strategy. Therefore, according to the expression of *Tbmstn1* and *Tbmstn2* in *T. bimaculatus*, we may speculate that these two genes may be related to the development of the skeletal muscle and embryos. However, the molecular mechanism of action derived from above and whether these two genes are involved in immune system and nervous system development are still unclear. As it is, further research is needed to continue.

## Conclusions

In this study, the full-length cDNAs of *Tbmstn1* and *Tbmstn2* were obtained. Both *Tbmstn1* and *Tbmstn2* had the highest expression in the skeletal muscle. Meanwhile, in embryogenesis, the expression of *Tbmstn1* started from the gastrula stage and was significantly higher in the eye-pigment formation stage and hatching stage than that in other stages. The *Tbmstn2* was expressed in all examined embryonic stages with different levels, and the highest expression was detected in the eye-pigment formation stage. These results suggested that *Tbmstn1* and *Tbmstn2* may be involved in the development of skeletal muscle and *Tbmstn2* may be related to the formation of nervous system. Their functions need to be further verified by molecular biological methods such as RNAi and gene overexpression in the future.

##  Supplemental Information

10.7717/peerj.9655/supp-1 Supplemental Information 1 qRT-PCR raw data for mstn1 in tissue

10.7717/peerj.9655/supp-2 Supplemental Information 2 qRT-PCR raw data for mstn2 in tissue

10.7717/peerj.9655/supp-3 Supplemental Information 3 qRT-PCR raw data for mstn1 in embryo

10.7717/peerj.9655/supp-4 Supplemental Information 4 qRT-PCR raw data for mstn2 in embryo

10.7717/peerj.9655/supp-5 Supplemental Information 5 mstn1 sequence

10.7717/peerj.9655/supp-6 Supplemental Information 6 mstn2 sequence

## Additional Information and Declarations

### DNA Deposition

The following information was supplied regarding the deposition of DNA sequences: The TbMSTN-1 and TbMSTN-2 cDNA sequences are available at NCBI GenBank: MN733728 and MN733729.

### Data Availability

The following information was supplied regarding data availability: Raw data is available in the Supplemental Files.
