# Supplementary material for: Characterization of two myostatin genes in pufferfish Takifugu bimaculatus: sequence, genomic structure, and expression
Source: PeerJ. 2020 Aug 3;8:e9655. doi: 10.7717/peerj.9655 (PMC7409809; doi:10.7717/peerj.9655)
Supplement: Supplemental Information 5 [file peerj-08-9655-s005.docx]

LOCUS MSTN-1 1461 bp mRNA linear VRT 25-NOV-2019

DEFINITION Takifugu bimaculatus.

ACCESSION MN733728

VERSION

KEYWORDS .

SOURCE Takifugu bimaculatus

ORGANISM Takifugu bimaculatus

Eukaryota; Metazoa; Chordata; Craniata; Vertebrata; Euteleostomi;

Actinopterygii; Neopterygii; Teleostei; Neoteleostei;

Acanthomorphata; Eupercaria; Tetraodontiformes; Tetradontoidea;

Tetraodontidae; Takifugu.

REFERENCE 1 (bases 1 to 1461)

AUTHORS wan,h.

TITLE Characterization of two myostatin genes in puffer fish Takifugu

bimaculatus: sequence, genomic structure, and expression pattern

JOURNAL unpublished

REFERENCE 2 (bases 1 to 1461)

AUTHORS wan,h.

TITLE Direct Submission

JOURNAL Submitted (25-NOV-2019) Jimei university, Fisheries College of

Jimei University, Jimei university, Xiamen city, Fujian province

361000, China

COMMENT Bankit Comment: ALT EMAIL:201714908003@jmu.edu.cn

Bankit Comment: TOTAL # OF SEQS:1

##Assembly-Data-START##

Sequencing Technology :: Sanger dideoxy sequencing

##Assembly-Data-END##

FEATURES Location/Qualifiers

source 1..1461

/organism="Takifugu bimaculatus"

/mol_type="mRNA"

/db_xref="taxon:433685"

CDS 164..1294

/codon_start=1

/product="MSTN-1"

/translation="MQLSPSMLHFSLMISLSLVVLSGQETHQQPPVGSPEDTEQCVTC DVRQHIKTMRLNAIKSQILSKLRMKEAPNISRDTVKQLLPKAPPLQQLLDQYDVLGDD NRDVVTEEDDEHAITETIMMMATEPASVVQLNGEPKCCHFSFTQKFQVSRLVRAQLWV HLRPAAEATTVFLQISRLMPVTDGNRHIRIRSLKLDVKAGASSWQSIDVKQVLSVWLR QPETNWGIEINAFDSRGKDLAVTSTQPGEEGLQPFMEVKISEGPRRVRRDLGLDCDEN SPESRCCRYPLTVDFEDFGWDWIIAPKRYKANYCSGECEYMHLQKYPHTHLVNKANPR GTAGPCCTPTKMSPINMLYFNQEQQIIYGKIPSMVVDRCGCL"

BASE COUNT 348 a 436 c 403 g 274 t

ORIGIN

1 caaaagcgac taaagttgga gtataaaaag gtgcgcgcta ataaagcatg atgcctctca

61 gtgtgggaca ttaatccaaa cccagcccag gctgcgcgtc agaaccagaa cacgccaagg

121 gatatacttt ttatagaacg catttccacg tcttcaggag acaatgcaac tgtctccgag

181 catgctgcat tttagcctga tgatttcttt gagtttggtg gtgttgagtg gccaagagac

241 gcaccagcag ccgcccgtcg gcagcccaga ggacacggag cagtgcgtca cctgcgacgt

301 ccgacagcac atcaaaacca tgcgattaaa cgccatcaag tctcagattc tgagcaagct

361 gcggatgaaa gaggctccga acatcagccg ggacacggtg aagcagctcc tgcccaaagc

421 gccgccgctg cagcagctcc tcgaccagta cgacgtgctg ggcgatgaca acagggacgt

481 ggtcacggag gaggacgacg agcacgccat cacggagacc atcatgatga tggccactga

541 acccgcgtcc gtcgtccaac tgaacgggga gcccaaatgc tgccatttct ccttcactca

601 aaagtttcaa gtcagccggc tggtccgcgc gcagctctgg gtgcatctgc gcccggcggc

661 cgaggccacc accgtgttcc tgcaaatctc ccgcctgatg ccggtcacag acgggaaccg

721 gcacatccgc atccgctccc tgaagctgga cgtgaaggcc ggggcgagct cctggcagag

781 catcgacgtc aagcaagtgc tgagcgtgtg gctgcggcag ccggagacca actggggcat

841 cgagattaac gcgttcgact cgagaggaaa agacttggcc gtgacctcca cgcagcccgg

901 agaggaaggc ctgcaacctt tcatggaggt gaagatctcg gaggggccca ggcgcgtccg

961 cagagacctg ggactggact gcgacgagaa ctctccagag tcccgttgct gccgctaccc

1021 gctcacggtg gactttgaag actttggctg ggactggatt atagccccaa agcgctacaa

1081 ggccaactat tgctccgggg agtgtgagta catgcacttg cagaaatacc cgcacaccca

1141 cctggtgaac aaggccaacc ccagagggac cgcgggccct tgctgtaccc ccaccaagat

1201 gtcgcccatc aacatgctct acttcaacca agaacagcag atcatctacg gaaagatccc

1261 ctccatggtg gtggaccgtt gtggatgtct ttaagtcggg gggccttcta gtgtcagact

1321 ttttgatgat ctaccagttc cagcgctttt tttactcctt cctgcagcga cacggtgcaa

1381 tagaaccaga gtagaggcct caaaccagtc cgaccttcct gcagggcaac accttatcag

1441 ctgccgtagc tctcactttc c

//
