# Supplementary material for: Characterization of two myostatin genes in pufferfish Takifugu bimaculatus: sequence, genomic structure, and expression
Source: PeerJ. 2020 Aug 3;8:e9655. doi: 10.7717/peerj.9655 (PMC7409809; doi:10.7717/peerj.9655)
Supplement: Supplemental Information 6 [file peerj-08-9655-s006.docx]

LOCUS MSTN-2 1211 bp mRNA linear VRT 25-NOV-2019

DEFINITION Takifugu bimaculatus.

ACCESSION MN733729

VERSION

KEYWORDS .

SOURCE Takifugu bimaculatus

ORGANISM Takifugu bimaculatus

Eukaryota; Metazoa; Chordata; Craniata; Vertebrata; Euteleostomi;

Actinopterygii; Neopterygii; Teleostei; Neoteleostei;

Acanthomorphata; Eupercaria; Tetraodontiformes; Tetradontoidea;

Tetraodontidae; Takifugu.

REFERENCE 1 (bases 1 to 1211)

AUTHORS wan,h.

TITLE Direct Submission

JOURNAL Submitted (25-NOV-2019) Jimei university, Fisheries College of

Jimei University, Jimei university, Xiamen city, Fujian province

361000, China

COMMENT Bankit Comment: ALT EMAIL:201714908003@jmu.edu.cn

Bankit Comment: TOTAL # OF SEQS:1.

FEATURES Location/Qualifiers

source 1..1211

/organism="Takifugu bimaculatus"

/mol_type="mRNA"

/db_xref="taxon:433685"

CDS 89..1168

/codon_start=1

/product="MSTN-2"

/translation="MLLLAVLTVVSAGFSMEMNQTSRLLAESGEQCSACDFREHSKQM RLHSIKSQILSILRLEQAPNISRDMIRQLLPKAPPLTQLLDQYDPRVEDEDHATTETI ITMATKPNPIAQDALSSCCLFSLSPKIQPKNILRALLWVHLRPADTVTTVFLQISRLK PGIEGNNTRVRVRSLRIDTDTAGAGSWQSVDIKSLLQAWLRQPETNYGIEINAFDSKG EDRAVTSLEPGEEGLQPFIEVKILNSPKRSRRESGLNCDEESAETRCCRYPLTVDFEE FGWDWIIAPKRYRANYCSGECEFLHLQQYPHAHLVNQANPRGTAGPCCTPTKMSPINM LYFNRKEQIIYGKIPSMVVDHCGCS"

BASE COUNT 293 a 380 c 312 g 226 t

ORIGIN

1 ctcctttatc agccattcat aaattcctga gttctggggc cggacccaga cggcgtgatc

61 acacagtgct gatcctcttc tctccaggat gctgctctta gctgtgctga ccgtcgtctc

121 tgcagggttt tccatggaga tgaaccagac ttccaggctg ctggcagaga gcggggagca

181 gtgctcggcc tgcgacttca gggagcacag caagcagatg aggctccaca gcattaagtc

241 ccagatcctg agcatactca gactggaaca ggctcccaac atcagccgag acatgatccg

301 ccagctgctg cccaaggcgc ctccgctcac gcagctcctg gaccagtacg atcccagggt

361 ggaggatgag gaccacgcca ccacggaaac catcataacc atggccacta agcctaatcc

421 catcgcccag gacgcgctgt cctcctgctg cctgttcagc ctcagtccga agatccagcc

481 caagaacatc ctgcgcgctc tgctgtgggt tcacctgcgg ccagctgaca ccgtcaccac

541 cgtctttctc cagatttccc gcctcaaacc tggaatagag ggaaacaaca cgcgggtcag

601 agtcagatcc cttcggattg acaccgatac ggccggtgct ggttcctggc agagcgtgga

661 catcaagtct ttgcttcaag cttggttgcg tcaaccagaa accaactacg gcattgagat

721 caacgccttc gattccaagg gagaagatcg agccgtcacc tcattagagc ctggagagga

781 agggttgcaa cccttcatcg aagtgaaaat cctcaacagc ccaaagagat cccgccgaga

841 atcgggcctc aactgcgacg aggaatccgc agaaacacgc tgctgtcggt acccgctcac

901 agtcgacttt gaggagtttg gatgggactg gatcatcgcg cccaagcgct accgggccaa

961 ctactgctca ggggagtgcg agttcctgca cctgcagcag tacccacatg cacacctggt

1021 gaaccaggcc aatccgaggg gcaccgcagg gccctgctgc acgcccacca agatgtcacc

1081 catcaacatg ctgtacttca atcgcaagga acagatcatc tacggaaaga tcccctcgat

1141 ggtggtcgac cactgcggct gctcctgaag acggtgaaaa tttcaacccc agcgaggaaa

1201 agaaatccca a

//
